# An Inhibitor of DRP1 (Mdivi-1) Alleviates LPS-Induced Septic AKI by Inhibiting NLRP3 Inflammasome Activation

**DOI:** 10.1155/2020/2398420

**Published:** 2020-07-11

**Authors:** Ruijin Liu, Si-cong Wang, Ming Li, Xiao-hui Ma, Xiao-nan Jia, Yue Bu, Lei Sun, Kai-jiang Yu

**Affiliations:** ^1^Department of Critical Care Medicine, The Harbin Medical University Cancer Hospital, Harbin, 150081 Heilongjiang Province, China; ^2^Department of Critical Care Medicine, The Second Affiliated Hospital of Harbin Medical University, Harbin, 150081 Heilongjiang Province, China; ^3^Department of Critical Care Medicine, The First Affiliated Hospital of Harbin Medical University, Harbin, 150001 Heilongjiang Province, China; ^4^Institute of Critical Care Medicine and Institute of Sino Russian Medical Research Center of Harbin Medical University, 150 Hapin Road, Harbin 150081, China

## Abstract

Mitochondria play an essential role in energy metabolism. Oxygen deprivation can poison cells and generate a chain reaction due to the free radical release. In patients with sepsis, the kidneys tend to be the organ primarily affected and the proximal renal tubules are highly susceptible to energy metabolism imbalances. Dynamin-related protein 1 (DRP1) is an essential regulator of mitochondrial fission. Few studies have confirmed the role and mechanism of DRP1 in acute kidney injury (AKI) caused by sepsis. We established animal and cell sepsis-induced AKI (S-AKI) models to keep DRP1 expression high. We found that Mdivi-1, a DRP1 inhibitor, can reduce the activation of the NOD-like receptor pyrin domain-3 (NLRP3) inflammasome-mediated pyroptosis pathway and improve mitochondrial function. Both S-AKI models showed that Mdivi-1 was able to prevent the mitochondrial content release and decrease the expression of NLRP3 inflammasome-related proteins. In addition, silencing NLRP3 gene expression further emphasized the pyroptosis importance in S-AKI occurrence. Our results indicate that the possible mechanism of action of Mdivi-1 is to inhibit mitochondrial fission and protect mitochondrial function, thereby reducing pyroptosis. These data can provide a potential theoretical basis for Mdivi-1 potential use in the S-AKI prevention.

## 1. Introduction

Sepsis has become the primary cause of AKI in patients. Infection in patients with chronic renal insufficiency often leads to further deterioration of renal function [1]. Currently, more attention is being paid to preventing AKI. Kidney replacement therapy is the only alternative when patients have severe electrolyte disorders, such as water and sodium retention, azotemia, hepatorenal syndrome, and other life-threatening pathophysiological changes [2].

The kidney receives 20% of the cardiac output. Nevertheless, its oxygen consumption corresponds to 10% of the body's oxygen consumption [3]. In addition, the kidney has the second highest mitochondrial content and oxygen consumption after the heart. Mitochondrial function can be severely affected and impaired by the compensatory range of cells and cellular damage caused by various factors, such as ischemia and hypoxia, toxin stimulation, heavy metal ions, and chemotherapy drugs. Thus, ATP synthesis decreases, causing a cell energy metabolism disorder and, subsequently, cell death [4]. Reactive oxygen species (ROS) produced by energy metabolism can initiate key cellular pathways, such as ROS/P38, ROS/Akt, and ROS/c-Jun N-terminal kinase (JNK). On the other hand, mitochondrial DNA (mtDNA) released by mitochondrial damage can trigger the mtDNA/interferon gene (STING) expression and stimulate the mtDNA/Caspase-1/3 signaling pathway, which are closely linked to increased cell death [5, 6].

Dynamin-related protein 1 (DRP1) is a cytoplasmic protein that can aggregate on the mitochondrial surface and induce mitochondrial division by interacting with mitochondrial fission protein 1 [7, 8]. Recent studies have shown that DRP1 inhibitors and knockout can significantly reduce renal ischemia-reperfusion injury in animal models [9]. Excessive mitochondrial division can lead to impaired cell function and increased release of apoptotic substances into mitochondria, which is detrimental to cells. Pyroptosis is characterized by lytic cell death Caspase-1 or Caspase-11 induced in mouse cells, which in turn leads to GSDMD-induced pore formation and cleavage of inflammatory procytokine interleukin-1*β* (IL-1*β*) and interleukin-18 (IL-18). Pyroptosis differs from other programmed cell death modes because it promotes the release of large amounts of proinflammatory factors that recruit a greater participation of inflammatory cells, thereby expanding the inflammatory response [10, 11]. This inflammatory pathway is widely found in macrophages, monocytes, and other immune cells, as well as in some nonimmune cells, including epithelial cells [12]. Our previous studies in animal models have shown that NOD-like receptor pyrin domain-3 knockout improves kidney function and inhibits inflammation in mice [13]. Several studies have proven that both ROS and mtDNA contribute to mitochondrial fever by activating the NLRP3 inflammasome [14]. Consequently, as per previous studies, by suppressing DRP1 expression, various mitochondrial functions have been improved, including increased mitochondrial membrane potential, increased mtDNA copy number, and decreased mitochondrial ROS [15]. Given this, we present a study to assess whether it is possible, by reducing the release of mitochondrial content, to protect mitochondrial function, inhibit pyroptosis, and consequently promote kidney function protection.

## 2. Materials and Methods

### 2.1. Animals

Male wild-type C57BL6 mice, 6-8 weeks old, weighing 20 to 25 g, taken from the River of Life laboratory in Beijing, were kept at constant temperature and humidity (22 ± 1°C and 50 ± 10%, respectively) and under a 12 h light/dark cycle in a laboratory animal care facility with free access to food and water. TheS-AKI mouse model was constructed by an intraperitoneal injection of 5 mg/kg lipopolysaccharide (LPS, *E. coli* O111: B4, Sigma, USA) according to previous reports [16]. Mdivi-1 (Sigma, USA) was properly dissolved in dimethyl sulfoxide (DMSO) and administered intraperitoneally at a dose of 3 mg/kg one hour before LPS injection. Mice were randomly assigned to 4 experimental groups (*n* = 6 per group): vehicle control group (mouse body weight of 5 mg/kg 0.9%saline + 3 mg/kg DMSO), vehicle Mdivi-1 group (5 mg/kg 0.9%saline + 3 mg/kg Mdivi − 1), S-AKI control group (5 mg/kg LPS + 3 mg/kg DMSO), and S-AKI+Mdivi-1 treatment group (5 mg/kg LPS + 3 mg/kg Mdivi − 1). After 24 hours, the mice were anesthetized with preconfigured mixed anesthetic, the eyeballs were removed, blood was collected, and the renal tissue was perfused and also collected. In order to ensure mouse stability, reduce the pain of the animals, and obtain analgesic and sedative effects, 80 mg/kg of ketamine (Jiangsu Hengrui, China) combined with 12 mg/kg of xylazine (Sigma, USA) was used as a mixed anesthetic. After total infusion with 0.9% saline, the right kidney of the mice was isolated and placed in 4% paraformaldehyde for 24 hours for hematoxylin-eosin (HE) staining. Each experimental group contained at least three right kidneys. The left kidney was divided into two lobes longitudinally centered on the renal hilum. The renal medulla was separated from the cortex, and after separation, a part of the renal cortex was placed at -80°C for Western blot and enzyme-linked immunosorbent assay (ELISA) analyses. The other part was placed in liquid nitrogen for mitochondrial separation and purification within 30 minutes. Each experimental group contained 6 left kidneys. The collected blood was placed in EDTA tubes and centrifuged at 4000 g for 15 min. The resulting plasma was stored at -80°C. The anesthesia and execution of mice followed the animal ethics standards of Harbin Medical University.

### 2.2. Cell Culture and Treatment

Mouse renal tubular epithelial (TCMK-1) cells, an immortalized cell line purchased from the American Type Culture Collection (ATCC), were cultured with DMEM/F12 containing 10% fetal bovine serum (Gibco, USA) in a constant temperature incubator at 37°C, 5% CO_2_, and 95% air. The S-AKI cell model was constructed by adding 5 *μ*g/ml LPS to the cell culture dish that was maintained for 12 hours. This model was already established and evaluated several times in the literature [17]. The Mdivi-1 (10 *μ*M) effect was evaluated in renal tubular epithelial cells, which were divided into four groups: vehicle control group, vehicle Mdivi-1 group, LPS control group, and LPS Mdivi-1 group. LPS was added 60 minutes after Mdivi-1 addition, and both were incubated together for another 12 hours. TCMK-1 cells were first inoculated into 6-well plates, and after the culture density reached 10^5^ per well, they were transfected with 8 *μ*l of blank adenoviral vector or siNLRP3 adenovirus for 4 hours. To reduce errors, pretests were performed in advance. The cultured cells were marked at different culture time points and counted using a cell counting plate to find the exact moment when the 10^5^ cell density was reached. For siRNA/siNLRP3 transfection experiments, the cells were washed three times with PBS, and fetal calf serum was added for further 48 hours of incubation. Cell viability was determined by a Cell Counting Kit 8 (CCK-8) assay kit (Beyotime, China) according to the manufacturer's protocol. Apoptosis analysis of cells was performed by flow cytometry and by using an Annexin V PE/7-AAD apoptosis detection kit (Solarbio, China). Annexin V-positive and 7-AAD-negative cells were defined as apoptotic cells.

### 2.3. Mitochondrial Function Determination

Mitochondria from kidney tissue and renal tubular epithelial cells were isolated using a Mitochondria Isolation Kit (Invitrogen, USA) according to the manufacturer's protocol. Isolated mitochondrial protein concentration was determined by the Bradford method. Mitochondrial function was defined by analyzing the mitochondrial membrane potential (*ΔΨ*m) and mitochondrial ROS content. *ΔΨ*m was measured in isolated mitochondria using the JC-1 (Invitrogen, USA). 100 *μ*g of purified mitochondria was added to the JC-1 staining solution in a 96-well plate for fluorescence determination, using 490 and 530 nm as excitation and emission wavelengths, respectively. The fluorescent measures were obtained in the SpectraMAX M5 reader, and the *ΔΨ*m was obtained by calculating the fluorescence units per milligram of protein. In cell experiments, *ΔΨ*m changes were observed by confocal microscopy.

Mitochondrial ROS activity was measured with MitoSOX Red (Invitrogen, USA), a redox-sensitive fluorescent probe that is selectively targeted to mitochondria. 50 *μ*g of isolated mitochondrial protein was incubated with MitoSOX solution (containing 5 *μ*M of the probe) for 30 minutes at 37°C and 5% CO_2_. The red fluorescence was determined using excitation and emission wavelengths of 510 and 580 nm, respectively, in a fluorescence plate reader. The data statistics are expressed in terms of fluorescence per milligram of protein.

### 2.4. Western Blot Analysis

The protein was separated by sodium dodecyl sulfate-polyacrylamide gel electrophoresis (8% SDS-PAGE). The protein in the gel was transferred to a polyvinylidene fluoride (PVDF) membrane by wet transfer, and 50 g/l skim milk was blocked for 2 hours. DRP1, gasdermin D (GSDMD), Caspase-1, NLRP3, and GAPDH antibodies (Abcam, USA) were incubated overnight at 4°C, and the membrane was washed 3 times with phosphate-buffered solution+Tween-20 (PBST). The corresponding secondary anti-rabbit antibody (1 : 10000) was incubated for 1 hour at room temperature, and the membrane was washed again with PBST. The detection of specific protein bands was performed by ECL, which were analyzed using ImageJ software.

### 2.5. Serum Creatinine (sCr) Determination

The determination of sCr in mice was performed using ELISA kits (Cloud Clone Corp, China) according to the manufacturer's instructions.

### 2.6. Determination of Malondialdehyde (MDA), Superoxide Dismutase (SOD), and Adenosine Triphosphate (ATP)

To detect the oxidative stress level in kidney tissue, colorimetric detection of MDA and SOD levels was performed by a kit (Beyotime Biotechnology, China) after homogenization of the renal tissue. In addition, the energy metabolism of cells was detected by determining the intracellular ATP level using a luciferase-based assay according to the manufacturer's instructions (Jiancheng, China).

### 2.7. HE Staining and Tubular Damage Score Measurement

Renal tissues were fixed with paraformaldehyde (4%) for 24 hours, paraffin-embedded, and sectioned at a thickness of 4 *μ*m. Then, they were processed for HE staining and evaluated using a light microscope (Olympus, Japan). The severity of the renal tubular injury was assessed using Paller et al.'s method [18].

### 2.8. GTPase Activity Assay of DRP1

To detect that the Mdivi-1 mechanism of action inhibits the DRP1 GTPase activity, instead of causing nonspecific effects of DRP1, the GTPase activity assay of DRP1 was performed in the control and Mdivi-1 treatment groups. First, the DRP1 protein was separated from TCMK-1 cells by immunoprecipitation. The Mdivi-1 treatment group received Mdivi-1 doses of 1, 5, and 10 *μ*M for 12 hours before protein extraction. The control group received a considerable volume of phosphate-buffered saline (PBS, Beyotime Biotechnology, China) solution and waited for the same time before protein extraction. After extracting the total cellular protein, 2 *μ*g of DRP1 antibody was added per 500 *μ*g of protein and rotated at 4°C overnight. Subsequently, 50 *μ*l of protein A/G-beads was added to different antigen-antibody mixtures and rotated at 4°C for 4 h. Then, this mixture was washed twice with RIPA lysis and extraction buffer and after was washed three times with GTPase buffer (2.5 *μ*M MgCl 2.50 *μ*M Tris-HCl pH 7.5). Subsequently, 0.5 mM GTP (Beyotime Biotechnology, China) was added and incubated at 30°C for 30 minutes. Finally, the GTPase activity measurement kit (Sigma, USA) was used according to the manufacturer's protocol, and the absorbance value obtained at 620 nm with a SpectraMAX M5 reader was identified as the relative DRP1 GTPase activity amount.

### 2.9. Statistical Analysis

The measurement data used are in the form of mean ± Standard Error of Mean (SEM). SPSS 21.0 statistical software was used for analysis. A one-way ANOVA was used to compare between groups. GraphPad Prism software (v10.0a) was used for drawing graphics. *P* < 0.05 was statistically significant.

## 3. Results

### 3.1. DRP1 Was Upregulated in LPS-Induced S-AKI Mouse Model

**Figure 1:**
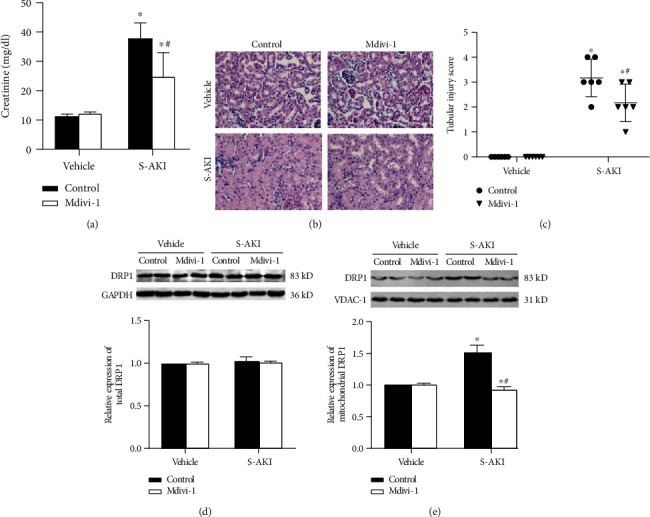
Mdivi-1 can alleviate kidney injury in mice. (a) Serum creatinine level in the vehicle control group (5 mg/kg 0.9%saline + 3 mg/kg DMSO), vehicle Mdivi-1 group (5 mg/kg 0.9%saline + 3 mg/kg Mdivi − 1), S-AKI control group (5 mg/kg LPS + 3 mg/kg DMSO), and S-AKI+Mdivi-1 treatment group (5 mg/kg LPS + 3 mg/kg Mdivi − 1). (b) Renal tubular injury score. The higher the score, the more severe the renal tubular injury. *n* = 6 mice in each group. (c) HE staining of the renal cortex observed under a light microscope (40x magnification). *n* = 3 − 6 mice in each group. (d) Western blot analysis of the DRP1 expression in the renal cortex. (e) DRP1 expression level in mitochondria after different treatments (*n* = 3 − 6 for each group).The data are presented as means ± SEM. ^∗^*P* < 0.05 versus control-treated mouse group. ^#^*P* < 0.05 versus LPS-induced S-AKI group (5 mg/kg LPS + 3 mg/kg DMSO).

Firstly, an S-AKI model was built in mice by intraperitoneal injection of LPS. The LPS application resulted in a significant increase in the creatinine level in septic mice (*P* < 0.05 for S-AKI control versus vehicle; Figure 1(a)). The degree of tubular edema and renal tubular injury scores showed by HE staining was significantly higher compared to that of the control group (Figures 1(b) and 1(c)). HE staining also shows that the renal tubules in the S-AKI group presented obvious edema, which was relieved after intervention with Mdivi-1. In addition, after Mdivi-1 injection in sepsis AKI mice, the serum creatinine level and renal tubular injury score decreased significantly (*P* < 0.05 for S-AKI+Mdivi-1 versus S-AKI control; Figures 1(a) and 1(c)). Western blot analysis showed there was no difference in DRP1 expression in whole cells between the different groups (*P* > 0.05 for S-AKI versus vehicle; Figure 1(d)). However, DRP1 expression in mitochondria was significantly upregulated in the S-AKI control group (*P* < 0.05 for S-AKI control versus vehicle; Figure 1(e)). Mdivi-1 administration improved both renal damage and tubular edema damage degrees (*P* < 0.05 for S-AKI+Mdivi-1 versus S-AKI control; Figure 1(e)). These data suggest that DRP1 is a S-AKI effector and that Mdivi-1 can partially alleviate kidney damage in S-AKI mice.

### 3.2. Mdivi-1 Downregulates NLRP3 Inflammasome Pathway Protein Expression and Protects Mitochondrial Function

**Figure 2:**
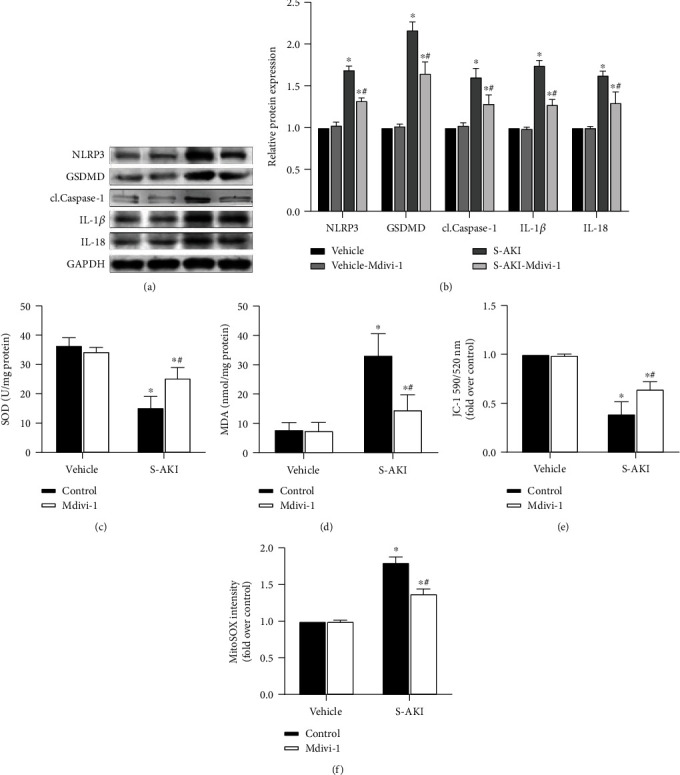
S-AKI mouse model shows that inhibition of DRP1 expression attenuates oxidative damage to kidney tissue and protects mitochondrial function by NLRP3 inflammasome pathway protein expression downregulation. (a) Western blot protein expression bands of the NLRP3 inflammasome-related proteins, NLRP3, GSDMD, cl.Caspase-1, IL-1*β*, and IL-18. (b) Semiquantitative analysis of Western blot protein expression bands of the NLRP3 inflammasome-related proteins. (c) Comparison of superoxide dismutase (SOD) levels in renal tissue homogenate between different treatment groups. (d) Comparison of malondialdehyde (MDA) levels in renal tissue homogenate between different treatment groups. (e) JC-1 staining of the purified mitochondria, which represents the mitochondrial membrane potential level in the different groups. (f) ROS staining of the purified mitochondria, which represents the reactive oxygen species level in mitochondria in the different groups. *n* = 6 mice in each group for all experiments. The data are presented as means ± SEM. ^∗^*P* < 0.05 versus control-treated mouse group. ^#^*P* < 0.05 versus LPS-induced S-AKI group.

The Mdivi-1 effects on the NLRP3 inflammasome protein expression in kidney tissue were assessed by Western blot analysis. The expression of NLRP3 inflammasome-related proteins (NLRP3, GSDMD, cl.Caspase-1, IL-1*β*, and IL-18) is significantly increased in the S-AKI group (*P* < 0.05 for S-AKI control versus vehicle; Figures 2(a) and 2(b)). Remarkably, Mdivi-1 administration decreases significantly the expression of these proteins (*P* < 0.05 for S-AKI+Mdivi-1 versus S-AKI control; Figures 2(a) and 2(b)). Oxidative stress damage of the renal tissue was also measured, and the MDA and SOD levels were higher in the S-AKI group (*P* < 0.05 for S-AKI control versus vehicle; Figures 2(c) and 2(d)). However, Mdivi-1 administration can partially alleviate oxidative damage in S-AKI mice (*P* < 0.05 for S-AKI+Mdivi-1 versus S-AKI control; Figures 2(c) and 2(d)). In addition, the mitochondrial function was evaluated by analyzing the mitochondrial membrane potential (*ΔΨ*m) and mitochondrial ROS content. *ΔΨ*m decreased and the MitoROS level increased in the LPS-induced group, but these effects were reduced after Mdivi-1 administration (*P* < 0.05 for S-AKI+Mdivi-1 versus S-AKI control; Figures 2(e) and 2(f)). These data show that inhibition of DRP1 expression reduces the NLRP3 inflammasome activation, which reduces the oxidative damage of kidney tissue in sepsis mice, possibly by protecting the mitochondrial function.

### 3.3. DRP1 Is Upregulated in LPS-Stimulated Renal Tubular Epithelial Cells

**Figure 3:**
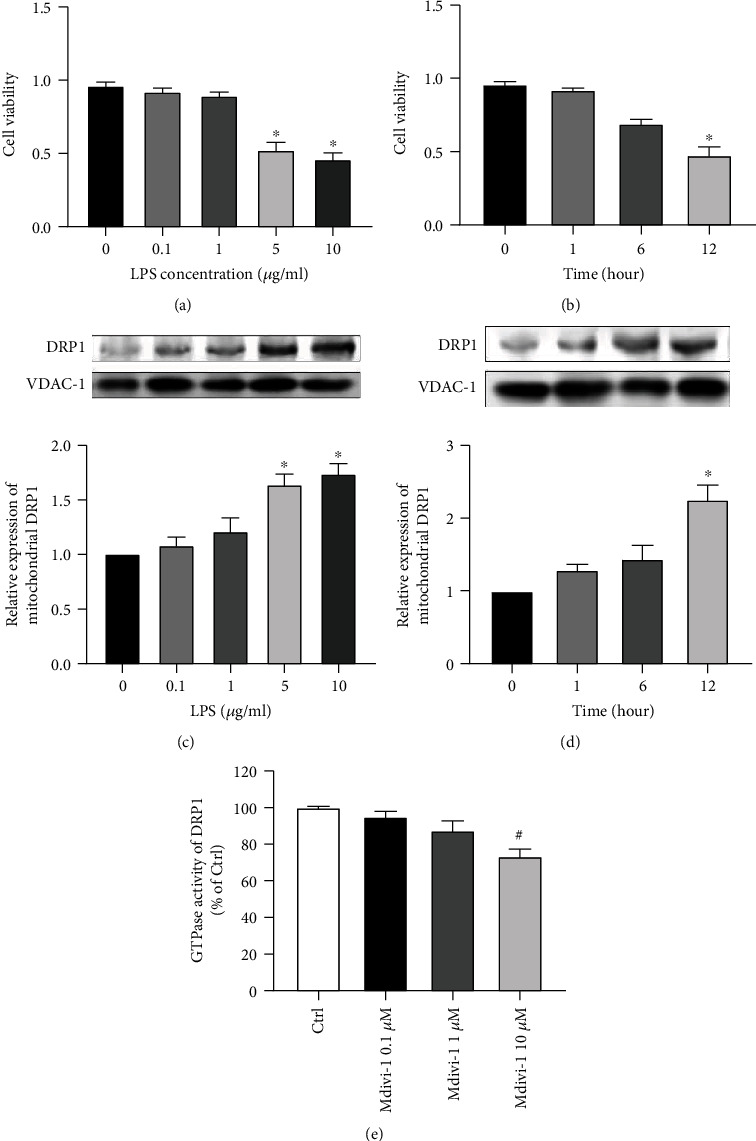
Lipopolysaccharide (LPS) treatment can induce the elevation of DRP1 expression in mouse renal tubular epithelial (TCMK-1) cells. (a) Cell viability was tested in the presence of concentrations of LPS (0, 0.1, 1, 5, and 10 *μ*g/ml) in 12 hours of culture. (b) Cell viability was tested in relation to different culture time points (0, 1, 6, and 12 hours). (c) Western blot assay of DRPI expression in mitochondria after different concentrations of administration of LPS (0, 0.1, 1, 5, and 10 *μ*g/ml) in 12 hours of culture. (d) Western blot assay of DRPI expression in mitochondria in different culture time points (0, 1, 6, and 12 hours) of 5 *μ*g/ml LPS administration. (e) GTPase activity assay of DRP1. *n* = 6 for each group in all experiments. The data are presented as means ± SEM. ^∗^*P* < 0.05 versus the concentration of LPS is 0 *μ*g/ml or versus time point is 0 hours. ^#^*P* < 0.05 versus control group.

A S-AKI cell model was constructed to further validate the critical role of DRP1 in the NLRP3 inflammatory pathway. For this, different time points in TCMK-1 cell culture and different LPS concentrations were tested to verify cell viability. Finally, 5 *μ*g/ml LPS (*P* < 0.05 versus the level of LPS is 0 *μ*g/ml; Figure 3(a)) and 12 hours of culture (*P* < 0.05 versus time point is 0 hours; Figure 3(b)) were the best parameters chosen. The mitochondrial DRP1 protein expression was detected by Western blot and showed that the dose and time point of LPS application were feasible (Figures 3(c) and 3(d)). In addition, it was evaluated whether Mdivi-1 could specifically inhibit DRP1 in HK-2 cells. Thus, the immunoprecipitated DRP1 protein was incubated with GTP, and a measurement kit was used to detect GTPase activity. It was found that the GTPase activity was significantly reduced when the concentration of Mdivi-1 was 10 *μ*M (*P* < 0.05 versus the control group; Figure 3(e)).

### 3.4. Mdivi-1 Downregulates the Expression of NLRP3 Inflammasome

**Figure 4:**
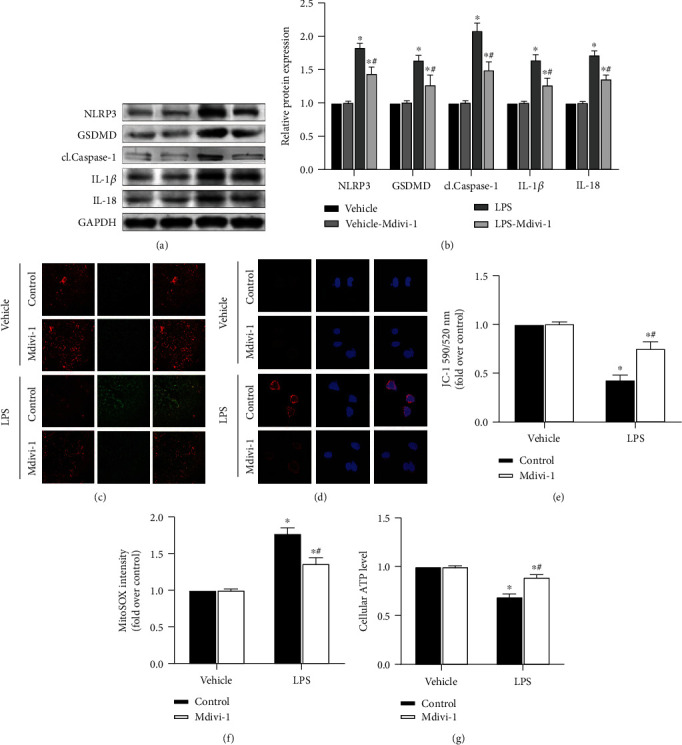
S-AKI cell model shows that DRP1 inhibition attenuates renal tubular epithelial pyroptosis and protects mitochondrial function by NLRP3 inflammasome pathway protein expression downregulation. (a) Western blot protein expression bands of the NLRP3 inflammasome-related proteins, NLRP3, GSDMD, cl.Caspase-1, IL-1*β*, and IL-18. (b) Semiquantitative analysis of Western blot protein expression bands of the NLRP3 inflammasome-related proteins. (c) JC-1 staining of cells, where the color red represents the membrane potential level. Image obtained under a confocal microscope (20x magnification). (d) MitoSOX staining of cells, where the color red represents the superoxidation level. Image obtained under a confocal microscope (60x magnification). (e) Quantitative analysis of JC-1 staining. (f) Quantitative analysis of MitoSOX staining. (g) Intracellular ATP levels in different treatment groups. *n* = 6 for each group in all experiments. The data are presented as means ± SEM. ^∗^*P* < 0.05 versus control-treated cells. ^#^*P* < 0.05 versus LPS-treated cells in the absence of Mdivi-1 treatment.

A 10 *μ*M dose of Mdivi-1 applied to renal tubular epithelial cells was able to significantly downregulate the expression of NLRP3-associated proteins compared to the LPS-stimulated group, as revealed by the Western blot assay (*P* < 0.05 for LPS-treated cells versus vehicle; Figures 4(a) and 4(b)). Analysis by confocal microscopy revealed that both *ΔΨ*m and green fluorescence on the confocal surface decreased after Mdivi-1 administration (*P* < 0.05 for LPS+Mdivi-1 versus LPS control; Figures 4(c) and 4(e)). In addition, fluorescent analysis showed that Mdivi-1 administration also induced a reduction in the MitoROS level (*P* < 0.05 for LPS+Mdivi-1 versus LPS control; Figure 4(d)) and in the red-stained material after MitoSOX Red assay (Figure 4(f)). Finally, intracellular ATP level measurement showed that the energy level increased after Mdivi-1 treatment (*P* < 0.05 for LPS+Mdivi-1 versus LPS control; Figure 4(g)). These data again indicate that pathway protein expression of the NLRP3 inflammasome can be attenuated by inhibiting DRP1 expression, possibly by protecting mitochondrial function.

### 3.5. siNLRP3 Downregulates NLRP3 Inflammasome Pathway Protein Expression and Apoptosis

**Figure 5:**
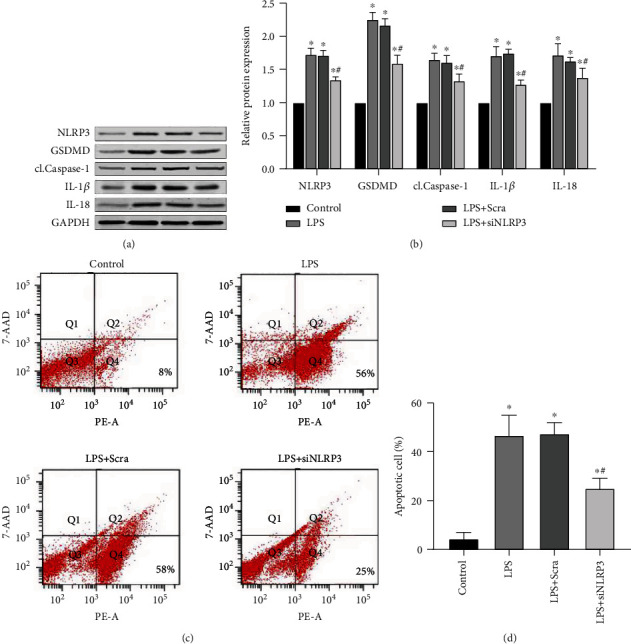
Knocking down the NLRP3 gene by siRNA/siNLRP3 transfection can attenuate the expression of pyroptosis pathway proteins and inhibit apoptosis. (a) Western blot protein expression bands of the NLRP3 inflammasome-related proteins, NLRP3, GSDMD, cl.Caspase-1, IL-1*β*, and IL-18 (*n* = 6 for each group). (b) Semiquantitative analysis of Western blot protein expression bands of the NLRP3 inflammasome-related proteins (*n* = 6 for each group). (c) Flow cytometry analysis of cell apoptosis. Q2 represents the number of cell necrosis and Q4 represents the number of apoptotic cells. The total number of cells is 10000. (d) Quantitative analysis of flow cytometry evaluation of cell apoptosis. Annexin V-positive and 7-AAD-negative (Annexin V^**+**^7-AAD^−^**)** cells were defined as apoptotic cells. The data are presented as means ± SEM. ^∗^*P* < 0.05 versus control-treated cells. ^**#**^*P* < 0.05 versus LPS-treated cell non-siNLRP3-transfected group.

To further validate the role of the NLRP3 inflammasome in cells, siRNA was transfected into renal tubular epithelial cells and the expression of NLRP3 inflammasome-related proteins was subsequently evaluated. Western blot assays showed that the expression of pyroptosis pathway proteins, NLRP3, GSDMD, cl.Caspase-1, IL-1*β*, and IL-18, was significantly reduced (*P* < 0.05 for LPS+siNLRP3 versus LPS-treated cells; Figures 5(a) and 5(b)). In addition, siNLRP3 transfection also decreased the amount of apoptotic cells, as detected by flow cytometry analysis (*P* < 0.05 for LPS+siNLRP3 versus LPS-treated cells; Figures 5(c) and 5(d)). These data indicate that inhibition of the NLRP3 gene expression can reduce apoptosis and that NLRP3 inflammasome-related proteins play an important role in LPS-induced pyroptosis.

## 4. Discussion

Mitochondria undergo great changes during AKI [19]. In particular, the early mitochondrial function cannot be corrected, resulting in mitochondrial function imbalances that may lead to cellular energy metabolism disorders and various pathophysiological changes. In recent years, in addition to cell necrosis and apoptosis, other programmed cell death pathways, such as ferroptosis, autophagy, and pyroptosis [20], have been found to play an essential role in the AKI pathogenesis. These cell death pathways are closely related to mitochondrial dysfunction. Therefore, a complete molecular understanding of mitochondrial damage has become an area of exploration for the development of novel therapies for AKI. As the first cause of AKI in ICU patients, S-AKI have attracted increasing attention in recent years [21].

The general dynamic characteristics of mitochondria are relatively well described. In a changing environment, mitochondria can react to different states and fuse into rod-like or ring-like structures. In these situations, the mitochondrial network remains tightly connected and the mitochondria are always in a dynamic equilibrium of fusion-division in almost all cells [22]. However, it is only in recent years that mitochondrial dynamics changes specifically in renal tubular epithelial cells have become more prominent [23], which may be related to the fact that most of mitochondrial dynamics have been performed on the fibrous skeleton of cardiomyocytes. Previous studies have shown that renal tubular epithelial cells contain a large number of mitochondrial fusion-division-related proteins [24]. It is speculated that mitochondrial fusion and division may also play an essential role in maintaining myocardial cell homeostasis. DRP1 is undoubtedly an essential protein in the process of mitochondrial division and fusion. ATP depletion in energy metabolism can lead to overactivation of DRP1, causing mitochondrial division [25, 26]. Drug-induced injury in the tubular epithelial cell model and ischemia-reperfusion injury model assays showed a sharp increase in DRP1 expression. At the same time, DRP1 inhibitor administration improves the mitochondrial function and reduces apoptosis [27]. Recent studies have shown that mitochondrial damage, including mitochondrial volume release, is related to several cell events, including autophagy, pyroptosis, or other programmed cell death mechanisms [28, 29], not limited to apoptosis.

Pyroptosis is a new type of cell death that differs from the other programmed cell death types in that it causes the release of inflammatory mediators. It is more classically activated by NLRP3 inflammatory bodies, causing downstream Caspase-1 cleavage and release of IL-1*β* and IL-18. Remarkably, excessive inflammatory factor release aggravates body injury [30].

In our study, we first constructed animal and cell models of S-AKI. DRP1 was overexpressed, and mitochondrial function was severely damaged in both models. The mitochondrial function damage includes a significant decrease in mitochondrial membrane potential and a large amount of mitochondrial ROS production. In addition, the MDA and SOD levels, which can reflect the oxidative damage of tissue, also increased significantly. Administration of Mdivi-1, a drug that has been reported to inhibit DRP1 [31], resulted in mitochondrial dysfunction protection and reduced expression of pyroptosis pathway proteins. Moreover, Mdivi-1 treatment promoted a reduction in creatinine levels and a significant improvement in the renal function of the animals. In addition, MDA and SOD levels also decreased significantly compared to the S-AKI group, indicating that the oxidative damage in kidney tissue was improved after Mdivi-1 treatment. Thus, we can draw a preliminary conclusion that improving mitochondrial function is useful for protecting renal function in mice. By protecting mitochondrial function, NLRP3 inflammasome-mediated pyroptosis is also significantly inhibited. We further demonstrated the critical role of pyroptosis in the S-AKI model by inhibiting the expression of NLRP3 inflammatory bodies through siNLRP3 transfection. After knocking down the NLRP3 gene, the expression of NLRP3 inflammasome-mediated pyroptosis pathway proteins, including NLRP3, GSDMD, Caspase-1, IL-1*β*, and IL-18, was downregulated to varying degrees. In addition, flow cytometry analysis showed that cell numbers that have undergone apoptosis were greatly reduced, highlighting the importance of this pathway.

It is undeniable that there are some points in this study that should be improved and considered. First, the treatment of S-AKI by Mdivi-1 is only partially repairing as it does not totally reduce the ROS production. Moreover, the antioxidant properties of Mdvi-1 described here are not the result of direct effects. Its mechanism of reducing tissue oxidative damage is to regulate mitochondrial fission-division by decreasing the DRP1 protein expression. Recently, some direct-acting compounds targeting mitochondria have been identified, such as MitoQ and Mito-TEMPO, which have proven to show a good antioxidant effect in the animal and cell models of AKI [32, 33]. Therefore, future studies may be performed to evaluate the antioxidant effects of the combination of Mdivi-1 with these compounds on AKI. In addition, although our results showed that Mdivi-1 administration can inhibit the GTPase activity of the DRP1 protein, a previous study has suggested that the inhibitory effect of Mdivi-1 on DRP1 can be nonspecific [34]. This study indicated that the inhibitory effect of Mdvi-1 is not achieved by affecting the GTPase activity but by inhibiting the complex I-dependent respiration-related pathway. Therefore, further studies are needed to verify whether Mdvi-1 can be used routinely for the DRP1 inhibition or whether it will be necessary to develop more efficient and specific inhibitors. Nevertheless, despite these limitations, we identified in this study a potential mechanism of reducing S-AKI by Mdivi-1 administration, which is based on mitochondrial function protection and NLRP3 inflammasome-mediated pyroptosis reduction.

## 5. Conclusion

DRP1 is highly expressed in the renal cortex of S-AKI animals, and after Mdivi-1 administration, its expression was significantly reduced. This Mdivi-1 effect may be due to the protection of mitochondrial function and the reduction of the NLRP3 inflammasome-mediated pyroptosis of renal tubular epithelial cells. This study suggests that Mdivi-1 may be a potential treatment or preventive agent for S-AKI.

## Data Availability

The data used to support the findings of this study are available from the corresponding author upon request.
