# Erratum to “An Inhibitor of DRP1 (Mdivi-1) Alleviates LPS-Induced Septic AKI by Inhibiting NLRP3 Inflammasome Activation”

**DOI:** 10.1155/2020/8493938

**Published:** 2020-09-19

**Authors:** Ruijin Liu, Si-cong Wang, Ming Li, Xiao-hui Ma, Xiao-nan Jia, Yue Bu, Lei Sun, Kai-jiang Yu

**Affiliations:** ^1^Department of Critical Care Medicine, The Harbin Medical University Cancer Hospital, Harbin, 150081 Heilongjiang Province, China; ^2^Department of Critical Care Medicine, The Second Affiliated Hospital of Harbin Medical University, Harbin, 150081 Heilongjiang Province, China; ^3^Department of Critical Care Medicine, The First Affiliated Hospital of Harbin Medical University, Harbin, 150001 Heilongjiang Province, China; ^4^Institute of Critical Care Medicine and Institute of Sino Russian Medical Research Center of Harbin Medical University, 150 Hapin Road, Harbin 150081, China

In the article titled “An Inhibitor of DRP1 (Mdivi-1) Alleviates LPS-Induced Septic AKI by Inhibiting NLRP3 Inflammasome Activation” [1], the published version of Figure 4(a) was a duplicate of Figure 2(a). This mistake was introduced during the production of the article, and the publisher apologises for this error.

The corrected figure is shown below and is listed as Figure 1.

## Figures and Tables

**Figure 1:**
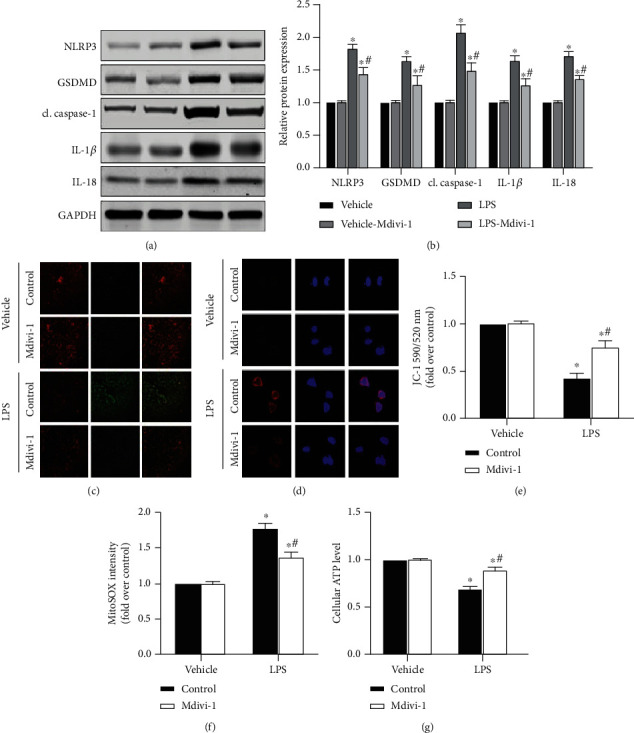
S-AKI cell model shows that DRP1 inhibition attenuates renal tubular epithelial pyroptosis and protects mitochondrial function by NLRP3 inflammasome pathway protein expression downregulation. (a) Western blot protein expression bands of the NLRP3 inflammasome-related proteins, NLRP3, GSDMD, cl. caspase-1, IL-1*β*, and IL-18. (b) Semiquantitative analysis of Western blot protein expression bands of the NLRP3 inflammasome-related proteins. (c) JC-1 staining of cells, where the color red represents the membrane potential level. Image obtained under a confocal microscope (20x magnification). (d) MitoSOX staining of cells, where the color red represents the superoxidation level. Image obtained under a confocal microscope (60x magnification). (e) Quantitative analysis of JC-1 staining. (f) Quantitative analysis of MitoSOX staining. (g) Intracellular ATP levels in different treatment groups. *n* = 6 for each group in all experiments. The data are presented as means ± SEM. ^∗^*P* < 0.05 versus control-treated cells. ^#^*P* < 0.05 versus LPS-treated cells in the absence of Mdivi-1 treatment.
